# The Impact of lncRNAs and miRNAs in Regulation of Function of Cancer Stem Cells and Progression of Cancer

**DOI:** 10.3389/fcell.2021.696820

**Published:** 2021-07-22

**Authors:** Soudeh Ghafouri-Fard, Mohammadreza Hajiesmaeili, Hamed Shoorei, Zahra Bahroudi, Mohammad Taheri, Guive Sharifi

**Affiliations:** ^1^Department of Medical Genetics, School of Medicine, Shahid Beheshti University of Medical Sciences, Tehran, Iran; ^2^Critical Care Quality Improvement Research Center, Shahid Beheshti University of Medical Sciences, Tehran, Iran; ^3^Department of Anatomical Sciences, Faculty of Medicine, Birjand University of Medical Sciences, Birjand, Iran; ^4^Department of Anatomical Sciences, Faculty of Medicine, Tabriz University of Medical Sciences, Tabriz, Iran; ^5^Urology and Nephrology Research Center, Shahid Beheshti University of Medical Sciences, Tehran, Iran; ^6^Skull Base Research Center, Loghman Hakim Hospital, Shahid Beheshti University of Medical Sciences, Tehran, Iran

**Keywords:** lncRNA, miRNA, cancer stem cell, expression, biomarker

## Abstract

Stem cells have two important features, namely the ability for self-renewal and the capacity to differentiate into some cell kinds with specialized functions. These two features are also present in cancer stem cells (CSCs). These cells have been detected in almost all kinds of cancers facilitating their tumorigenicity. Molecular cascades that control self-renewal of stem cells, namely the Wnt, Notch, and Hedgehog pathways have been suggested to influence CSCs functions as well. Moreover, non-coding RNAs can regulate function of CSCs. Function of miRNAs in the regulation of CSCs has been mostly assessed in breast cancer and hepatocellular carcinoma. miR-130a-3p, miR-600, miR-590-5p, miR-142-3p, miR-221, miR-222, miR-638, miR-375, miR-31, and miR-210 are among those regulating this feature in breast cancer. Moreover, miR-206, miR-192-5p, miR-500a-3p, miR-125, miR-125b, miR-613, miR-217, miR-194, and miR-494 regulate function of CSCs in hepatocellular carcinoma. DILC, lncTCF7, MUF, HAND2-AS1, MALAT1, DLX6-AS1, HOTAIR, and XIST are among lncRNAs that regulate function of CSCs. In the present paper, we explain the effects of these two classes of non-coding RNAs in the regulation of activity of CSCs.

## Introduction

Stem cells have two important features, namely the ability for self-renewal and the capacity to differentiate into some cell kinds with specialized functions. The latter capacity enables them to produce more stem cells that are kept in an undifferentiated status. However, the latter feature permits production of mature cell types. Several lines of evidence has showed the existence of a group of cells with stem-like features inside tumors (Yu et al., 2012). Being designated as cancer stem cells (CSCs), these cells show features of both stem cells and cancer cells. They not only have self-renewal and differentiation abilities, but also they can give rise to tumors when transferred into an animal (Yu et al., 2012). The primary indication pointing to the presence of CSCs came from Lapidot et al. (1994) study showing organization of human acute myeloid leukemia as a hierarchy that is derived from primitive hematopoietic cells (Lapidot et al., 1994). After nearly a decade, this model was adapted to breast cancer when Al-Hajj et al. (2003) showed the presence of a tumorigenic subpopulation capable of induction of cancer in NOD-SCID mice. This population was characterized by CD44+/CD24−/low feature (Al-Hajj et al., 2003). Subsequent studies have verified the presence of CSCs in other types of cancers as well (Singh et al., 2004). Functionally, signaling cascades that control self-renewal of stem cells, namely the Wnt, Notch, and Hedgehog pathways have been reported to influence CSCs functions as well (Rosen and Jordan, 2009). CSCs features are thought to be affected by several factors among them are certain genetic abnormalities and the stage of cancer progression (Rosen and Jordan, 2009). Meanwhile, function of CSCs is controlled by a number of non-coding RNAs, particularly long non-coding RNAs (lncRNAs) and microRNAs (miRNAs). Both lncRNAs and miRNAs can affect development of cancer through modulation of important cancer-related pathways such as Notch (Ghafouri-Fard et al., 2021c), Rho-GTPase (Ghafouri-Fard et al., 2021d) and NF-κB (Ghafouri-Fard et al., 2021a) pathways, activity of immune-related cascades (Ghafouri-Fard et al., 2021b) and epithelial-mesenchymal transition (EMT) (Hussen et al., 2021). In the present paper, we explain the effects of these two classes of non-coding RNAs in controlling function of CSCs and their impact on the tumorigenesis.

## miRNAs and CSCs

### Breast Cancer

miR-130a-3p is a putative tumor suppressor miRNA whose expression has been found to be diminished in human breast cancer samples and blood-derived exosomes. Forced up-regulation of miR-130a-3p in breast CSCs has suppressed proliferation, migratory potential, and invasiveness, while its silencing has led to opposite effects. Functionally, miR-130a-3p decreases expression of RAB5B. Besides, down-regulation of exosome-originated miR-130a-3p has been correlated with involvement of lymph nodes and advanced clinical stage. Based on these results, miR-130a-3p has been suggested as a biomarker for monitoring breast cancer progression and a target for treatment of breast cancer (Kong et al., 2018). miR-600 is another miRNA whose silencing enhances expansion of breast CSCs, whereas its up-regulation decreases self-renewal of these cells, resulting in attenuation of tumorigenicity in animal models. miR-600 has been shown to target SCD1 which codes a protein with essential role in the production of active, lipid-altered WNT proteins. Up-regulation of miR-600 suppresses generation of active WNT and increases differentiation of breast CSCs. Down-regulation of miR-600 in clinical samples has been associated with activation of WNT signaling and poor clinical outcome (El Helou et al., 2017). miR-590-5p is another miRNA which decreases breast CSC population. This miRNA inhibits expression of SOX2. Experiments in NOD/SCID mice have shown that miR-590-5p inhibits tumorigenicity of breast cancer cells (Zhou et al., 2017). Table 1 shows the role of miRNAs in regulation of breast CSCs.

**TABLE 1:** Role of miRNAs in regulation of breast CSCs (ANTs, adjacent non-cancerous tissues; BCa, breast cancer).

microRNA	Samples	Cell lines	Target	Function	References
miR-130a-3p	40 pairs of cancerous and ANTs, blood samples from BCa and healthy controls (*n* = 40)	Breast cancer stem cells (BCSCs), MCF-7, 293T, MDA-MB-231, MCF-10A,	RAB5B, EGFR	miR-130a-3p by regulating RAB5B inhibits invasion and migration in human BCSC-like cells.	Kong et al., 2018
miR-600	Mouse/Human; BCa (*n* = 120)	Breast cancer stem cells (bCSCs), SUM149/Basal, SUM159/Mesenchymal, S68/Luminal	SCD1, GSK3-β, GSK3-α, Wnt/β-catenin	miR-600 via SCD1 by influencing the WNT/β-catenin pathway could regulate BCSC Fate.	El Helou et al., 2017
miR-590-5p	Mouse/Human; 49 pairs of cancerous and ANTs	MCF-7, ZR75-1	SOX2	miR-590-5p by targeting SOX2 could inhibit breast cancer cell stemness and metastasis.	Zhou et al., 2017
miR-142-3p	–	MDA-MB-468, HCC1806, MCF-7	BRCA1, BRCA2, β-catenin, Bod1, KLF4, Oct4	miR-142-3p by reducing β-catenin could attenuate breast CSC characteristics and radioresistance.	Troschel et al., 2018
miR-221, miR-222	Mouse	MCF-7, MDA-MB-231	PTEN, COX-2, ALDH1, p65, AKT, NF-kB	miR-221/222 by targeting PTEN via activating the AKT/NF-κB/COX-2 pathway could promote tumor growth and cancer stem-like cell properties.	Li et al., 2017
miR-638	Mouse/Human; 60 pairs of cancerous and ANTs	MCF-10A, MCF-7	E2F2, SOX2, OCT4,	miR-638 by targeting E2F2 could repress the characteristics and behaviors of breast CSCs.	Lin Q.-Y. et al., 2020
miR-375	–	MCF-7	HOXB3, CD44, CD133, MTDH, TWIST	miR-375 by targeting HOXB3 could inhibit CSCs phenotype and resistance to tamoxifen in human ER-positive BCa.	Fu H. et al., 2017
miR-31	Mouse/TCGA database	Mammary stem cell (MaSC), Mammary epithelial cells	Axin1, GSK-3β, Smad2/3, Smad4, LBH, p21, ITGA2, ITGB1, Gata3, K14/18, ERα, Cyclin-D1, c-Myc, β-casein, P63, p65, Ikkα, RANKL, NF-κB, Prlr/Stat5, TGFβ, Wnt/β-catenin	miR-31 via suppressing the Wnt signaling antagonists could promote breast tumorigenesis and MaSC expansion.	Lv et al., 2017
miR-210	Mouse/Human; BCa (*n* = 8)	MCF-7, 293T, MDA-MB-231	*E*-cadherin, Snail	Up-regulation of miR-210 via targeting *E*-cadherin could promote proliferation, self-renewal of CSCs and metastasis.	Tang et al., 2018
miR-200c/141	Mouse/Human; 25 pairs of cancerous and ANTs	MCF-7, BT474, T47D, 293T	HIPK1, β-catenin, *E*-cadherin, Snail, Vimentin, Zeb1, YWHAG	miR-200c/141 by targeting the HIPK1/β-catenin axis could regulate breast CSC heterogeneity.	Liu et al., 2018a
miR-155	Mouse/Human; 38 pairs of cancerous and ANTs	MDA-MB-231	ABCG2, CD44, CD90, CD24	miR-155 could act as a therapeutic target for BCa, preventing cancer stem cell formation.	Zuo et al., 2018
miR-1976	Mouse/Human, TCGA database, 35 pairs of TNBC and ANTs	SUM-1315-br, SUM-1315-bo, MDA-MB-231, ZR-75-1, MCF-7	PIK3CG, *E*-cadherin, *N*-cadherin, Slug, Vimentin, Snail, CD44, AKT	Knockdown of miR-1976 by PIK3CG could promote CSCs properties and EMT in TNBC.	Wang J. et al., 2020

### Hepatocellular Carcinoma (HCC)

Expression of miR-206 has been found to be diminished in chemoresistant and recurrent HCC tumors. Notably, expression of this miRNA has also been reduced in CD133 or EpCAM-positive hepatic CSCs and in CSC-enriched spheres in hepatoma. Forced up-regulation of miR-206 has been shown to inhibit expansion of hepatic CSCs through blocking dedifferentiation of hepatoma cells and decreasing self-these cells. Mechanistically, EGFR has been described to be targeted by miR-206 (Liu et al., 2020). Expression profile of 14 miRNAs has been found to be altered among five groups of CSC-positive HCC tissues, namely EpCAM+, CD90+, CD133+, CD44+, and CD24+ HCC samples. Among these miRNA, miR-192-5p has been identified as the most important miRNA being under-expressed in all five groups of CSC-positive HCC samples, while being abundant in liver tissues. miR-192-5p silencing has facilitated expansion of CSC populations and CSC-associated characteristics via influencing expression of PABPC4. TP53 mutations and excessive methylation of the promoter area of this miRNA have been identified as underlying mechanisms of inactivation of miR-192-5p transcription in HCC cells and primary CSC-positive HCC (Gu et al., 2019). On the other hand, expression of miR-500a-3p has been significantly increased in HCC tissues and related cell lines. Over-expression of miR-500a-3p has been correlated with poor outcome of these patients. Over-expression of miR-500a-3p has enhanced the spheroid formation capacity, proportion of side population and levels of CSC markers. Besides, this miRNA has increased *in vivo* tumorigenicity of HCC cells. Functionally, miR-500a-3p enhances CSC features through influencing expression of numerous negative modulators of JAK/STAT3 signaling, such as SOCS2, SOCS4, and PTPN11, resulting in constituent activation of STAT3 cascade (Jiang et al., 2017a). Table 2 shows the role of miRNAs in regulation of HCC CSCs.

**TABLE 2:** Role of miRNAs in regulation of CSCs in hepatocellular carcinoma (ANTs, adjacent non-cancerous tissues; HCC, hepatocellular carcinoma).

microRNA	Samples	Cell lines	Target	Function	References
miR-206	Tissue samples of HCC (*n* = 40)	Liver cancer stem cells (CSCs), Huh7, HepG2, Hep3B, CSQT-2, PLC, HCCLM3, L02	EGFR, CD133, Nanog, Sox-2, OCT4, c-Myc, Bmi-1, β-catenin, PARP	miR-206 by targeting EGFR could suppress liver CSCs expansion.	Liu et al., 2020
miR-192-5p	Mouse/Human; HCC Cohort 1 (*n* = 241), Cohort 2 (*n* = 372), Cohort 3 (*n* = 53)	HuH1, HuH7, Hep3B, SK-Hep1, HLE, HLF, 293T	PABPC4, CD133, EpCAM, CD44, CD24, CD90, UGT2B7, GSTA1, CYP1A2	miR-192-5p by targeting PABPC4, and genetic aberrations could suppress the CSC-related features in HCC.	Gu et al., 2019
miR-500a-3p	Mouse/Human; 8 pairs of cancerous and ANTs	97H, HepG2, QGY-7703, Hep 3B, PLC, Huh 7, SMMC7721, L02	SOCS2, SOCS4, SOCS6, PTPN11, PTPN4, JAK/STAT3	miR-500a-3p via STAT3 pathway could promote cancer stem cells properties in HCC.	Jiang et al., 2017a
miR-125, miR-125b	HCC (*n* = 6)	TAMs, Huh7, HepG2, BEL-7404	CD90, CD81, CD63, CD44, MMP9, Snail1	miR-125a/b-mediated down-regulation of CD90 has essential effects in CSCs of HCC.	Ge et al., 2019
miR-613	Mouse/Human	Huh7, HCCLM3	SOX9, PARP	miR-613 by targeting SOX9 could inhibit liver CSCs expansion and HCC cell dedifferentiation.	Li et al., 2019
miR-217	Mouse/Human/TCGA database; 6 pairs of cancerous and ANTs	97H, HepG2, QGY-7703, Hep3B, PLC, Huh7, SMMC7721, L02	DKK1, P84, NANOG, BMI-1, OCT4, SOX2, WNT	miR-217 by targeting DKK1 via activating the Wnt pathway could increase CSC properties in HCC.	Jiang et al., 2017b
miR-194	Mouse/Human; 110 pairs of cancerous and ANTs	Huh7, HCCLM3, LM3, PLC, hepG2, hep3B, 97L, 97H, Human primary hepatoma cells	RAC1, PARP, MKK4, P38, *N*-cadherin, HBEGF, PTPN12	miR-194 by regulating RAC1 could suppress expansion of CSCs.	Ran et al., 2019
miR-494	Mouse/Human; Tumor and cirrhotic tissues (*n* = 75)	Huh7, HepG2, SNU449, SNU182	p27, PTEN, Sox2, DNMT3B, OCT4, CDKN2B, BBC3, PROM1, Caspase-3, PARP, AKT/mTOR	miR-494 via AKT/mTOR pathway could regulate stem-cell phenotype and increases sorafenib resistance in HCC.	Pollutri et al., 2018
miR-296-5p	Mouse/Human; 89 pairs of cancerous and ANTs	MHCC97H, Huh7	Brg1, Sall4, EpCAM, CD90, Nanog, Sox2, Klf4, Oct4	miR-296-5p via the Brg1/Sall4 axis could inhibit stem cell potency of HCC cells.	Shi et al., 2020

### Colorectal Cancer (CRC)

Unrestrained proliferation of cancer cells has been shown to induce hypoxia within the tumor mass, supporting activity of CSCs through induction of certain hypoxia-responsive routes. Expansion of CSCs in hypoxic conditions is associated with high sphere and colony construction. miR-215 has been identified as one of the principal hypoxia-associated miRNAs in primary colon CSCs. miR-215 is a negative modulator of CSC-enhancing influences of hypoxia. LGR5 has been acknowledged as a downstream molecule in hypoxia/miR-215 cascade. This miRNA has a prominent tumor suppressive effect in CRC and a target for anti-CSCs modalities (Ullmann et al., 2019).

Comparison of miRNA profile between CSC-enriched CRC cells (EpCAM+/CD44+) and CSC-depleted cells has led to identification of miR-221 as the most abundantly expressed miRNA in EpCAM+/CD44+ CRC cells. Over-expression of miR-221 has been associated with expression of Lgr5 in mouse colon crypts and poor clinical outcome of CRC in human subjects. Constitutive up-regulation of miR-221 has increased organoid-forming ability of both CRC cell lines and patient-originated xenograft *in vitro*. Notably, suppression of miR-221 has inhibited these features. QKI-5 has been identified as miR-221 target (Mukohyama et al., 2019).

miR-92a is another CSC-related miRNA which has been found to be over-expressed in chemoresistant CRC cell lines and tissues. Forced up-regulation of miR-92a has enhanced resistance to cytotoxic effects of 5-fluorouracil on CRC cells, while its silencing has remarkably promoted chemosensitivity *in vivo*. Most notably, up-regulation of miR-92a has increased tumor sphere construction and enhanced expression of stem cell biomarkers. miR-92a has been reported to activate Wnt/β-catenin signaling through inhibiting KLF4, GSK3β, and DKK3, which negatively regulate Wnt/β-catenin signaling at multiple levels. Besides, IL-6/STAT3 cascade has been shown to increase miR-92a levels through targeting its promoter, leading to activation of Wnt/β-catenin cascade and subsequent enhancement of stem-like features in CRC (Zhang G.-J. et al., 2017). Table 3 shows the role of miRNAs in regulation of CSCs in CRC.

**TABLE 3:** Role of miRNAs in regulation of CSCs in CRC (ANTs, adjacent non-cancerous tissues).

microRNA	Samples	Cell lines	Target	Function	References
miR-215	Mouse/Human; 50 pairs of cancerous and ANTs	Primary spheroid cultures T6, T18, T20, T51, primary colon tumor-initiating cell	LGR5	miR-215 by targeting LGR5 could counteract hypoxia-induced CSC activity.	Ullmann et al., 2019
miR-221	Mouse/Human/TCGA database; CRC (*n* = 6), colon normal (*n* = 4)	HCT116, 293T, EpCAMÞ^+^/CD44^+^, EpCAMÞ/CD44, PDX-KUC1	QKI-5	miR-221 by targeting QKI can enhance the malignant abilities of human CRC stem cells.	Mukohyama et al., 2019
miR-92a	Mouse/Human; tumor tissues from CRC responding (*n* = 12) and non-responding (*n* = 12)	HT-29, HCT116, HT29/5-FU, HCT116/5-FU	KLF4, GSK-3β, DKK3, PARP, CD133, SOX2, OCT4, MYC, CCND1, MMP-7, Wnt/β-catenin, IL6/STAT3	IL-6/STAT3/miR-92a/Wnt/β-catenin axis could regulate stem cell-like properties in CRC.	Zhang G.-J. et al., 2017
miR-450a-5p	Mouse/Human; 90 pairs of cancerous and ANTs	SW480, SW620, 293T, HUVECs	SOX2, CD133, CD31, V-cadherin, *E*-cadherin, Nango, Snail, Twist, Vimentin	miR-450a-5p by targeting SOX2 could regulate cancer stem cell properties and angiogenesis in CRC.	Wang M. et al., 2020

### Gastric Cancer

Gastric CSCs are described by expression of the stem cell marker CD44. CD44(+) cells have been shown to form more sphere colonies and pose higher level of invasiveness compared with gastric cancer cells lacking this marker. One of the supreme up-regulated miRNAs in gastric CSCs is miR-196a-5p. Inhibition of expression of miR-196a-5p has reduced colony formation and invasiveness of gastric CSCs. Functionally, miR-196a-reduces Smad4 levels through targeting its 3′-UTR. Smad4 levels in gastric cancer tissues have been associated with levels of differentiation, TNM stage and deepness of invasion. Notably, up-regulation of Smad4 has abolished miR-196a-5p-associated EMT in CSCs (Pan et al., 2017). miR-26a has been shown to be down-regulated in gastric cancer. This miRNA targets HOXC9, an up-regulated gene and a prognosticator of poor clinical outcome in gastric cancer. Over-expression of miR-26a in gastric cancer cells has suppressed HOXC9 levels and inverted its effects on self-renewal of CSCs (Peng et al., 2018). miR-7-5p is another down-regulated miRNA in gastric CSCs. Notably, expression of this miRNA has been enhanced in the methionine-deprived medium. Excessive DNA methylation in the promoter region has been found to be the main cause of down-regulation of miR-7-5p in gastric cancer. Up-regulation of miR-7-5p has decreased colony formation and invasiveness of gastric CSCs via targeting Smo and Hes1 and consequent suppression of Notch and Hedgehog cascades. Up-regulation of miR-7-5p has suppressed growth of gastric cancer in the animal models (Xin et al., 2020). Table 4 shows the role of miRNAs in gastric CSCs.

**TABLE 4:** Role of miRNAs in regulation of gastric CSCs (ANTs, adjacent non-cancerous tissues).

microRNA	Samples	Cell lines	Target	Function	References
miR-196a-5p	95 pairs of GC and ANTs	Gastric cancer stem cell (GCSCs), SNU-5, BGC-823	Smad4, Sox2, Oct4, Nanog, CD44, *N*-cadherin, Vimentin, slug, Snail, *E*-cadherin	miR-196a-5p by targeting Smad4 could modulate GCSCs characteristics.	Pan et al., 2017
miR-26a	Mouse/Human/GEO database; 52 pairs of GC and ANTs	BGC823, MKN45, MKN28, SGC7901, 293T	HOXC9, *E*-cadherin, *N*-cadherin, Vimentin, MMP2, MMP7, Twist1, Zeb1, Snail1, SOX2, SOX4, Oct4, β-catenin	Dysregulation of miR-26a/HOXC9 could promote stem cell-like features and metastasis.	Peng et al., 2018
miR-7-5p	Mouse	BGC-823, SGC-7901, gastric cancer stem cells (GCSCs)	Smo, Hes1, c-myc, CD44, Sox2, Oct4, Nanog, Notch, Hedgehog	miR-7-5p via suppressing Hes1 and Smo could suppress GCSCs invasion.	Xin et al., 2020
miR-132	Mouse/Human; 20 pairs of GC and ANTs	Gastric cancer stem cell (GCSCs), MKN45, MKN28	SIRT1, CREB, ABCG2	miR-132 in Lgr5+ GCSCs-like cells via regulating SIRT1/CREB/ABCG2 pathway could contribute to cisplatin-resistance.	Zhang L. et al., 2017

### Glioma

Ramakrishnan et al. (2020) have profiles miRNA signature of glioblastoma samples before and after radiotherapy. All assessed samples had unmethylated MGMT promoter and wild-type IDH (Ramakrishnan et al., 2020). MGMT acts as a DNA “suicide” repair enzyme. The encoded protein amends impaired guanine nucleotides through transporting the methyl at O6 site of guanine to its cysteine residues, therefore preventing gene mutation, apoptosis and carcinogenic processes induced by alkylating agents (Yu et al., 2020). Although expression of most of miRNAs did not change after treatment, expression of a number of miRNAs reduced. miR-603 has been identified as the most altered miRNA. This miRNA has been found to target IGF1 and IGF1R. Cellular transfer of miR-603 via release of extracellular vesicles has been increased following exposure with ionizing radiation, leading to de-repression of IGF1 and IGF1R and enhancement of expansion of CSCs and acquired resistance to radiotherapy. Moreover, miR-603 export has de-repressed MGMT (Ramakrishnan et al., 2020).

Expression of miR-223 has been increased in glioma tissues. Up-regulation of miR-223 has increased survival of these cells treated with Temozolomide (TMZ), while its suppression reversed this feature. PAX6 has been recognized to be targeted by miR-223. miR-223/PAX6 axis has been shown to regulate growth, invasiveness, and resistance TMZ via modulating PI3K/Akt signaling (Liu et al., 2017a).

The Chinese traditional medicine, Bufalin has been shown to inhibit proliferation, colony construction, and CSC features and enhance cell apoptosis and expression of miR-203 by glioblastoma cells. Notably, up-regulation of miR-203 results in similar effects as treatment with bufalin. SPARC has been acknowledged as a target of miR-203. Taken together, miR-203 mediates to effects of bufalin in suppression of growth of glioma cells and CSCs development (Liu et al., 2017b). *SPARC* gene codes for a cysteine-rich acidic matrix-associated protein which has essential role in the biogenesis of extracellular matrix, induction of alterations to cell shape and invasiveness of tumor cells (Brekken et al., 2003). Table 5 summarizes the role of miRNAs in regulation of glioma CSCs.

**TABLE 5:** Role of miRNAs in regulation of glioma CSCs (ANTs, adjacent non-cancerous tissues).

microRNA	Samples	Cell lines	Target	Function	References
miR-603	Mouse/Human; progressive disease (*n* = 14) or stable disease (*n* = 12)	LN340, A1207, LN18, T98G, U87MG, BT-147, BT-99, CMK3, BT-83	IGF1R, MGMT, Olig2, TSG101, HSC70, ApoA1, CD9, CD81	miR-603 via downregulation of IGF1 could suppress GBM stem-cell state.	Ramakrishnan et al., 2020
miR-223	20 pairs of GBM and peritumoral brain edema (PTBE) tissue	Glioblastoma stem cell (GSCs), U251	PAX6, CD113, Nestin, PI3K/AKT	miR-223 by targeting PAX6 via regulating PI3K/AKT pathway could regulate GSCs proliferation and the chemoresistance to temozolomide (TMZ).	Liu et al., 2017a
miR-124	Human	U87, 293T	CDK6	Delivery of Exogenous miR-124 by WJ-MSCs could decrease Cell Proliferation and confers chemosensitivity in GBM cell.	Sharif et al., 2018
miR-203	-	U251, U87	SPARC, OCT4, SOX2.	Bufalin by upregulation of miR-203 could inhibit cancer stem cell-like phenotype and cellular proliferation in Glioma.	Liu et al., 2017b
miR-150-5p	Mouse/Human; 29 pairs of Glioma and adjacent normal brain tissues	U87, U251, U373, A172, NHA	c-MYC, LEF1, SOX9, Oct-4, CCND1, CD133, CXCR4, ABCG2, Nango, Nestin, Wnt/β-catenin	miR-150-5p by targeting the Wnt/β-catenin pathway could suppress the stem cell-like characteristics of glioma cells.	Tian et al., 2020

### Leukemia

miR-378 is an up-regulated miRNA in chronic myeloid leukemia (CML) patients compared with normal persons. Up-regulation of miR-378 has enhanced proliferation and drug-resistance of CML cells, while inhibiting their apoptosis. Notably, transfection of cell with miR-378 has led to expansion of stem cell spheres and up-regulation of OCT4 and c-Myc. Moreover, miR-378 suppresses levels of FUS1 (Ma et al., 2019), a tumor suppressor that activates intrinsic apoptotic pathway via induction of Apaf-1-related mechanisms and hinders activities of protein tyrosine kinases (Ji and Roth, 2008). A high throughput transcriptome analysis of treatment-naive CML stem/progenitor cells has led to identification of miR-185 as a predictor of response to ABL tyrosine kinase inhibitors (TKIs). miR-185 has a tumor suppressive effect. Forced over-expression of miR-185 has impaired survival of drug-resistant cells, enhanced their response to TKIs, and noticeably eradicated long-term repopulating leukemic stem cells and infiltrating blasts. These effects have been accompanied by higher survival of xenotransplantation models. miR-185 has been shown to target PAK6. Suppression of PAK6 expression has interfered with activity of the RAS/MAPK pathway and mitochondria, enhancing sensitivity of resistant cells to TKIs (Lin H. et al., 2020). Table 6 shows the role of miRNAs in regulation of leukemic CSCs.

**TABLE 6:** Role of miRNAs in regulation of leukemic CSCs.

microRNA	Samples	Cell lines	Target	Function	References
miR-378	CML (*n* = 59)	Bone marrow mononuclear cells (BMMNCs), K562	FUS1, OCT4, c-Myc	miR-378 via enhancing stem cell properties could promote leukemia K562 cell proliferation.	Ma et al., 2019
miR-185	Mouse/Human; CML cohort 1 (*n* = 22) and cohort 2 (*n* = 58), healthy group (*n* = 11)	Bone marrow stem cell (hBMSCd), K562, K562R, BV173, UT-B/A, UT-B/A-T315I	PAK6, ABL1, STAT5, CRKL, RAS/MAPK	miR-185 by targeting PAK6 could regulate survival of drug-resistant leukemic stem cells.	Lin H. et al., 2020

### Other Cancers

In renal cell carcinoma, expression of miR-106b-5p has been found to elevated compared with normal controls. Up-regulation of miR-106b-5p in these cells has enhanced spheres formation capability and the quantity of side population cells. Further experiments in an orthotopic renal cancer model and a tail vein injection have shown that up-regulation of miR-106b-5p enhances tumor growth and metastasis to lungs, respectively. miR-106b-5p has been recognized as an activator of Wnt/β-catenin signaling which instantaneously inhibits numerous negative regulators of this pathway, including LZTFL1, SFRP1, and DKK2 (Lu et al., 2017b).

miR-328-3p is an up-regulated miRNA in ovarian CSCs. Over-expression of miR-328 results in better maintenance of CSC features through affecting expression of DNA damage binding protein 2, a protein that suppresses activity of ovarian CSCs. Attenuation of activity of ERK pathway in ovarian CSCs contributes in up-regulation of miR-328 and expansion of CSCs. miR-328 silencing in mouse orthotopic ovarian xenograft has blocked tumor growth and suppressed metastatic ability (Srivastava et al., 2019).

miR-335 has been revealed to be down-regulated in osteosarcoma CSCs compared with differentiated cells. Up-regulation of miR-335 has been associated with reduced stem cell-like features. On the other hand, miR-335 silencing has inhibited stem cell-like features and invasiveness. POU5F1 has been shown to be targeted by miR-335. The inhibitory effects of miR-335 on CSCs has exerted a synergic effect with traditional chemotherapeutic substances in the treatment of osteosarcoma (Guo et al., 2017). Since POU5F1 is an essential regulator of pluripotency (Nichols et al., 1998), miR-335 can affect pluripotency of stem cells through regulating its expression.

Expression of miR-1275 has been increased in lung cancer cell lines and tissues. Up-regulation of miR-1275 in clinical samples has been associated poor clinical outcome. Expression of miR-1275 is induced by the proto-oncogene HIF-1α. miR-1275 enhances the activity of Wnt/β-catenin and Notch pathways and consequently increases the stemness of lung adenocarcinoma cells. miR-1275 can suppress expression of multiple negative regulators of Wnt/β-catenin and Notch pathways, namely DKK3, SFRP1, GSK3β, RUNX3, and NUMB (Jiang et al., 2020). Table 7 shows the impact of miRNAs on CSCs in different cancers.

**TABLE 7:** Impact of miRNAs on CSCs in other cancers (ANTs, adjacent non-cancerous tissues).

Type of cancer	microRNA	Samples	Cell lines	Target	Function	References
Clear cell renal cell carcinomas (ccRCC)	miR-106b-5p	Mouse/Human, TCGA database, 20 pairs of ccRCC and ANTs	786-O, A498, HK-2, ACHN, OSRC-2, Caki-1/2, 769-P	LZTFL1, SFRP1, DKK2, HDAC1, Wnt/β-catenin	miR-106b-5p via activating the Wnt/β-catenin signaling could promote RCC aggressiveness and stem-cell-like phenotype.	Lu et al., 2017b
Ovarian cancer (OC)	miR-328–3p	Mouse	OVCAR4, SKOV3, OV2008	DDB2, Sox2, Nanog, Oct4, ERK1/2	Disruption of the ROS/ERK/miR-328/DDB2 axis could impair CSC function and prevent metastasis in OC.	Srivastava et al., 2019
Osteosarcoma (OS)	miR-335	Mouse	MG63, U2OS, 143B, 293T	POU5F1, Sox2	miR-335 by targeting POU5F1 could regulate OS stem cell-like properties.	Guo et al., 2017
OS	miR-26a	Mouse/Human; tumor samples (*n* = 53)	U2OS, MG63, Saos-2, 143B, ZOS, ZOSM	Jagged1, Notch, OCT3/4, NANOG, SOX2, c-Myc nucleostemin (NS), CD133, NCID, HES1	miR-26a/Jagged1/Notch axis could inhibit stem cell-like phenotype and tumor growth of OS.	Lu et al., 2017a
Lung Adenocarcinoma (LUAD)	miR-410	Mouse/Human	Human umbilical cord mesenchymal stem cells (hUCMSCs), H1299, PC-9	PTEN, CD63, calreticulin, LATS2, HOXA5, Smad4, KLF 10	The hUCMSC-derived extracellular vesicles by transferring miR-410 could promote LUAD growth.	Dong et al., 2018
LUAD	miR-1275	Mouse/Human; 196 pairs of LUAD and ANTs	EBAS-2B, L78, H460, A549, GLC-82, SPC-A1, PC9, H1299, H1975, H2228	DKK3, SFRP1, GSK-3β, RUNX3, NUMB, β-catenin, NICD, HISTH3, Wnt/β-catenin, Notch	HIF-1α-regulated miR-1275 via activating Wnt/β-catenin and Notch could promote the progression of LUAD and maintains stem cell-like features.	Jiang et al., 2020
Non-small cell lung carcinoma (NSCLC)	miR-708-5p	Mouse/Human/TCGA database; NSCLC (*n* = 148)	A549, Calu-3, 95D	DNMT3A, Dnmt3a, Dnmt3b, p21, 5-mC, CDH1, CD34, D133, Wnt/β-catenin	miR-708-5p by targeting DNMT3A via repressing Wnt/β-catenin signaling could inhibit lung cancer stem cell-like phenotypes.	Liu et al., 2018c
NSCLC	has-mir-485-5p	Mouse/Human; Serum samples from NSCLC (*n* = 16), normal persons (*n* = 15)	A549, H460, H1299, 293T	RXRα, CD133, CD44, Sox2, Nanog, Oct4, MMP-9, *E*-cadherin,	Epigallocatechin-3-gallate (EGCG) by modulating the hsa-mir-485-5p/RXRα axis could inhibit CSC-like properties.	Jiang et al., 2018
NSCLC	miR-181b	Mouse/Human/TCGA database; 8 pairs of NSCLC and ANTs	H1650, H1299, A549, A549/DDP	Notch2, Hes1, KLF4, SOX2, NANOG, CD133, ALDH, Caspase-3, Bcl-2, NICD2, HEY1, PARP, PARP	miR-181b by targeting Notch2 could regulate CSC-like properties, and overcome chemoresistance in NSCLC.	Wang et al., 2018
Leukemia/Lymphoma syndrome	miR-339	Mouse	BBC2, KG1, BaF3, NIH3T3, 293T	BCL-2L11, Bax, FGFR1	miR-339-5p via downregulation BCL-2L11 and Bax could promote development of Stem cell leukemia/lymphoma (SCLL) syndrome.	Hu et al., 2018
Prostate cancer (PCa)	miR-1301-3p	PCa (*n* = 136), Normal (*n* = 22)	N1, N2, 22Rv1, C4-2B, DU-145, LNCap, TSU-PrI, PC-3, α-tubulin, P84	SFRP1, GSK-3β, OCT4, SOX2, NANOG, CD44, KLF4, c-MYC, MMP2, β-catenin, AKT	miR-1301-3p by targeting GSK-3β and SFRP1 via activating the Wnt pathway could promote expansion of prostate CSCs.	Song et al., 2018

## lncRNAs and CSCs

Contribution of lncRNAs in the expansion of CSCs has been verified in several investigations. A number of lncRNAs have been reported to regulate this phenotype in different types of cancers. MALAT1, HOTAIR, and XIST are among the mostly assessed lncRNAs in this regard.

### MALAT1

MALAT1 expression has been revealed to be increased in cancer spheroids compared with parental liver cancer cells. MALAT1 silencing has decreased sphere construction and reduced expression of stem cell markers in liver cancer cells. The effects of MALAT1 on CSCs are mediated through sponging miR-375, and subsequently up-regulation of YAP1 (Zhao et al., 2020), a protein that regulates expression of genes contributing in cell proliferation and blocks expression of apoptotic genes (Sudol, 1994). Thus, MALAT1/miR-375/YAP1 represents a functional axis in liver carcinogenesis and a target for elimination of CSCs (Zhao et al., 2020). Similarly, in glioma cells, MALAT1 silencing has inhibited expression of two stemness-related factors, namely Sox2 and Nestin (Han et al., 2016). Since Nestin regulates assembly and disassembly of intermediate filaments (Guérette et al., 2007), MALAT1-mediated regulation of Nestin can affect cell remodeling. In these cells, MALAT1 mainly regulates activity of ERK/MAPK pathway (Han et al., 2016).

### HOTAIR

HOTAIR is another lncRNA which enhances expansion of CSCs. In liver cancer cells, HOTAIR increases stemness features via suppressing expression of SETD2. From a mechanistical point of view, HOTAIR decreases the recruitment of the CREB, P300, RNA polII on promoter of *SETD2* gene, reducing its expression and phosphorylation. Thus, the ability of SETD2 for binding with substrate histone H3 is attenuated and trimethylation of lysine 36th of histone H3 is decreased, leading to reduction of H3K36me3-hMSH2-hMSH6-SKP2 complex. Notably, the complex tenancy on chromosome is decreased and mismatch DNA repair function is reduced (Li et al., 2015). Consistent with this study, HOTAIR silencing in CD133(+) CRC CSCs has significantly reduced the tumor growth and metastatic ability in xenograft model of CRC (Dou et al., 2016). In oral squamous cell carcinomas, HOTAIR has been found to be up-regulated in tumor samples, particularly in the metastatic ones. HOTAIR silencing has remarkably suppressed stemness, invasiveness and tumorigenicity in xenografts. On the other hand, up-regulation of HOTAIR has led to promotion of metastatic ability and EMT. Notably, HOTAIR levels have been positively correlated with levels of mesenchymal markers and negatively related with epithelial markers (Lu et al., 2017c).

### XIST

Expression of XIST has been increased in glioma tissues and CSCs. XIST silencing has reduced cell proliferation, migratory potential and invasiveness, while increasing apoptosis. XIST knock down has also inhibited *in vivo* growth of tumor and enhanced survival of affected animals. Mechanistically, XIST interacts with miR-152. Through modulation of this miRNA, XIST regulates functions of glioma stem cells (Yao et al., 2015). In bladder cancer, XIST sponges miR-200c and enhances clone formation, self-renewal aptitude and EMT in bladder CSCs (Xu et al., 2018).

### GAS5

GAS5 is a tumor suppressor lncRNA with acknowledged roles in several tissues (Ji et al., 2019). In pancreatic cancer cells, GAS5 regulates chemoresistance to gemcitabine and metastatic ability of transformed cells. Up-regulation of GAS5 could suppress proliferation, migratory potential, resistance to gemcitabine, stem cell-like features, and EMT process through sequestering miR-221 and releasing SOCS3 from its inhibitory effects. Moreover, GAS5 could enhance effects of gemcitabine on suppression of tumor growth and metastasis. The GAS5/miR-221/SOCS3 cascade has been identified as an important modulator of EMT and CSC function in pancreatic cancer (Liu et al., 2018b).

### DILC

lnc-DILC has an acknowledged role in suppression of expansion of CSCs, since its silencing has led to enhancement of expansion of liver CSCs and induction of initiation and progression of liver cancer. This lncRNA can inhibit autocrine activity of IL-6/STAT3 cascade through directly binding with *IL-6* promoter. Besides, lnc-DILC can mediate the interaction between TNF-α/NF-κB pathway and IL-6/STAT3 axis. Consistent with these findings, expression of lnc-DILC has been found to be reduced in patients with liver cancer. Moreover, expression of lnc-DILC has been correlated with IL-6, EpCAM or CD24 levels. Down-regulation of lnc-DILC in these patients has been correlated with risk of early recurrence and poor clinical outcome. Thus, lnc-DILC can connect liver inflammation with expansion of CSCs through mediating the interplay between mentioned pathways (Wang X. et al., 2016). Table 8 demonstrates the role of lncRNAs in CSCs.

**TABLE 8:** Role of lncRNAs in CSC (ANTs, adjacent normal tissues).

Type of disease	LncRNA	Samples	Cell lines	Target	Function	References
Hepatocellular carcinoma (HCC)	DILC	Mouse/Human; 195 pairs of HCC and ANTs	Liver cancer stem cells (LCSCs), Huh7, HepG2, CSQT-2	IL-6, STAT3, TNF-α, NF-kB, JAK2	lnc-DILC via IL-6/STAT3 axis could regulate LCSCs.	Wang X. et al., 2016
HCC	lncTCF7	Mouse/Human; 30 pairs of cancerous and ANTs	Cancer stem cells (CSCs), Huh7, Hep3B	EEA1, Sox2, Nanog, C-myc, Oct4, BAF170, BRG1, SNF5, WNT	lncRNA lncTCF7 via activation of Wnt signaling could promote self-renewal of human liver CSCs.	Wang et al., 2015
HCC	MUF	Mouse/TCGA database	HCC-associated mesenchymal stem cells (HCCMSC), 293T, Hep3B, PLC, Huh7, HepG2, MHCC-97L, HCCLM3, SMMC-7721	miR-34a, ANXA2, *E*-cadherin, FN, Snail1, GSK-3b, Vimentin, Wnt/β-catenin	Mesenchymal stem cells via the MUF/miR-34a/ANXA2 axis could promote HCC.	Yan et al., 2017
HCC	HAND2-AS1	Mouse/Human; 8 pairs of cancerous and ANTs	Hep3B, Huh7, PLC/PRF/5	BMP, INO80, RUVBL2, BMPR1A, SMAD1/5, IES2, HAND2, Digoxin, AMIDA, ARP4, ARP5, BAF53A, IES6, YY1, GFP	HAND2-AS1 via BMP signaling could promote liver CSCs self-renewal.	Wang et al., 2019
HCC	MALAT1	15 pairs of cancerous and ANTs	Huh7, Hep3B, HepG2, 293T	miR-375, YAP1, c-Myc, Oct4, Sox2	MALAT1 via sponging the miR-375/YAP1 axis could modulate CSC properties of HCC.	Zhao et al., 2020
HCC	DLX6-AS1	Mouse/Human/TCGA database; 48 pairs of cancerous and ANTs	Liver cancer stem cells (LCSCs), SMMC-7721, HCCLM3, Hep3B, HepG2, Huh7, L02	CADM1, OCT-4, SOX2, Nanog, STAT3	Downregulation of DLX6-AS1 by CADM1 via inactivating of the STAT3 pathway could inhibit the stem cell properties of LCSCs.	Wu et al., 2019
Liver cancer	HOTAIR	Mouse/Human; 65 pairs of liver cancer and ANTs	Human liver cancer stem cell (hLCSC)	SETD2, CREB, P300, RNA polII, H3k36me3, Skp2, Cyclin-D1, Cyclin-E, CDK2, CDK4, ppRB, E2F1, PCNA	HOTAIR via downregulation of SETD2 could promote malignant growth hLCSC.	Li et al., 2015
Glioblastoma (GBM)	XIST	Mouse/Human; Normal brain tissues (NBTs) (*n* = 8), Glioma tissues Grade I (*n* = 8), Grade II (*n* = 8), Grade III (*n* = 8), Grade IV (*n* = 8)	Human glioblastoma stem cells (GSCs), 293T	miR-152, IgG, Ago2	Knockdown of XIST via upregulating miR-152 could promote apoptosis of GSCs.	Yao et al., 2015
GBM	TP73-AS1	GBM (*n* = 33)	Glioblastoma cancer stem cells (gCSC), G26, G7, 293T	ALDH1A1	TP73-AS1 could promote TMZ resistance in gCSC and tumor aggressiveness.	Mazor et al., 2019
Glioma	MALAT1	Human	Glioma stem cell line SHG139S, SHG139	Sox2, Nestin, OCT-4, CD133, Nanog, A2B5, ERK/MAPK	Downregulation of MALAT1 could regulate expression of stemness markers.	Han et al., 2016
Pancreatic Ductal Adenocarcinoma (PDAC)	HOTTIP	Mouse/Human; PDAC (*n* = 90)	Pancreatic cancer stem cells (PCSCs), PANC-1, SW1990	HOXA9, LIN28, NANOG, OCT4, Sox2, WDR5, C-myc, Cyclin-D1, β-tubulin, Lin28, Nanog, Wnt/β-catenin	HOTTIP via regulating HOXA9 modulate PCSC in PDAC.	Fu Z. et al., 2017
Pancreatic cancer (PC)	GAS5	Mouse/Human; 60 pairs of PC and ANTs	HPDE6-C7, PANC-1, AsPC-1, Capan-2, SW1990, BXPC-3	miR-221, SOCS3, *E*-cadherin, *N*-cadherin, Vimentin, Snail, Oct4, CD133, Nanog, Sox2	GAS5 via targeting miR-221/SOCS3 could reverse CSC-mediated resistance to gemcitabine and EMT in PC.	Liu et al., 2018b
Prostate cancer	lnc-ROR	Mouse	Human prostate cancer stem cells (CD44^+^ /CD133^+^ HuPCaSCs), Du145, 22RV1	miR-145, Ccnd1, Cdk4, Oct4, CD44, CD133	Curcumin by ceRNA effect of ROR and miR-145 could suppress proliferation and invasion of HuPCaSCs.	Liu et al., 2017a
Colorectal cancer (CRC)	cCSC1	Mouse/Human; 52 pairs of CRC and ANTs	NCM460, Caco2, SW480, SW620, LoVo, HT29, HCT116	Gil1, SMO, Sox2, CXCR4, CD133, CD44, Nanog, Hedgehog	cCSC1 via activating of the Hedgehog pathway could modulate cancer stem cells (CSCs) properties in CRC.	Zhou et al., 2020
CRC	BCAR4	Mouse/Human; 30 pairs of CRC and ANTs	HCT116, HCT8, SW480, CCD 841 CoN, 293T	miR-665, STAT3, ALDH, CD133, Sox2, Nanog, Oct4, CD44, Lgr5, BCAR4	BCAR4 via miR-665/STAT3 axis could promote tumorigenicity in CCR and maintains cancer stem cell stemness.	Ouyang et al., 2019
CRC	HOTAIR	Mouse	LoVo, cancer stem cells (CD133^+^ CSCs)	Vimentin, *E*-cadherin, *N*-cadherin	Downregulation of HOTAIR could inhibit the invasiveness and metastasis of CRC cancer stem cells.	Dou et al., 2016
Colon cancer	MBNL1-AS1	Mouse/TCGA database	HT29	miR-412-3p, MYL9, CD133, PCNA, HMGB1, Bcl-2, Bax	Overexpression of MBNL1-AS1 via inhibiting miR-412-3p could inhibit CSC-related invasion and migration.	Zhu et al., 2020
Gastric cancer (GC)	ADAMTS9-AS2	Mouse	Cancer stem cells (CSCs), MKN45	SPOP, Oct3/4, Sox2, Nanog, CD44	ADAMTS9-AS2 by regulating SPOP could suppress the tumorigenicity of CSCs in GC.	Wang F. et al., 2020
GC	ROR	GC (*n* = 33)	Gastric cancer stem cell (GCSCs), MKN-45	Oct4, Sox2, Nanog, CD133	ROR could regulate the invasion, proliferation, and stemness of GCSCs.	Wang S. et al., 2016
Nasopharyngeal cancer (NPC)	PVT1	15 pairs of NPC and ANTs	5-8F, HNE1, HNE2, CNE1, CNE2	miR-1207, Oct4, c-Myc, Sox2, ALDH, PI3K/AKT	Pvt1 by inhibiting miR-1207 via activating PI3K/AKT pathway could promote CSC–like traits in NPC.	Cui et al., 2019
Oral squamous cell carcinomas (OSCC)	HOTAIR	Mouse/Human/TCGA database; Non-cancerous matched tissues (*n* = 15), paired tissue samples from the tumor (*n* = 15), metastatic lymph nodes (*n* = 15)	Oral carcinomas stem cells (OCSC), NHOK, Fadu, OECM-1, GNM, Ca9-22, SCC4, HSC3	*E*-cadherin, Vimentin, FN-1, SNAIL1, TWIST, ZEB1s, Snail, Oct4, Nanog, SOX2	HOTAIR silencing via modulation of EMT could suppress stemness and metastasis in OCSC.	Lu et al., 2017c
Cervical cancer (CC)	CCAT1	39 pairs of CC and ANTs	SiHa, HeLa, CaSki, HCC94, C33A, H8, SFCs	miR-185-3p, FOXP3	SOX2/lncRNA CCAT1/miR-185-3p/FOXP3 Axis could promote the self-renewal of CC stem cells.	Zhang et al., 2021
Triple-negative breast cancer (TNBC)	NRAD1	Mouse/TCGA Database	SUM149, MCF7, MDA-MB-468, BT20, HCC70, HCC1599, HCC38, SKBR3, HCC1143, BT549, HCC1937, MDAMB453, MDAMB468	–	NRAD1 acts in downstream of ALDH1A3.	Vidovic et al., 2020
Papillary thyroid carcinoma (PTC)	DOCK9-AS2	Mouse/Human; 54 pairs of PTC and ANTs	Nthy-ori3-1, BCPAP, TPC1	miR-1972, CTNNB1, CD63, CD81, TSG-101, Alix, Calnexin, CD133, *E*-cadherin, *N*-cadherin, MMP2/7, Nanog, Oct4, SOX2, EpCAM, ALDH1A1, GSK-3β, c-Myc, H3, SP1, Wnt/β-catenin	CSCs-derived exosomal DOCK9-AS2 via activating the Wnt/β-catenin pathway could regulate proliferation, invasion, and stemness in PTC cells.	Dai et al., 2020
Melanoma	LHFPL3-AS1	Mouse	Melanoma stem cells, MDA-MB-435, 293T	miR-181a-5p, Bcl-2	LHFPL3-AS1 via the miR-181a-5p/BCL-2 pathway could participate in tumorigenesis of melanoma stem cells.	Zhang et al., 2020
Multiple myeloma (MM)	MEG3	MM (*n* = 6), Normal (*n* = 3)	MSCs, 293T	BMP4, Runx2, Osx, OCN, SOX2, Smad1/5/8	Upregulation of MEG3 by targeting BMP4 could promote osteogenic differentiation of MSCs from MM Patients	Zhuang et al., 2015
Breast cancer (BC)	Hh	Mouse	MCF-7, Hs578T, BT549, MDA-MB-231, MCF10A, 293T, Mammosphere	GLI1, SOX2, Oct4, Twist, ALDH1, *E*-cadherin, Histone H3, Vimentin, SMAD3, SMAD1/5/8, Erk, Hedgehog	Hh via activating Hedgehog pathway could play a role in mediating Twist-induced CSC properties.	Zhou et al., 2016
Osteosarcoma (OS)	HIF2PUT	30 pairs of OS and ANTs	U2OS, MG-63, hFOB	HIF2	HIF2PUT via regulating HIF2 could inhibit osteosarcoma stem cell proliferation and invasion.	Zhao et al., 2019
Bladder cancer	XIST	Mouse	Human bladder cancer stem cell (BCSC), 5637, T24	miR-200c, KLF4, Oct4, ABCG2, *E*-cadherin, Vimentin, CD133, CD44, ZEB1/2	XIST by targeting miR-200c could regulate stemness features and tumorigenicity of human BCSCs.	Xu et al., 2018

## Discussion

Cancer stem cells have especial properties that potentiate them for anti-cancer treatment, since it is expected that treatments targeting these cells preclude cancer metatstasis and recurrence. Therefore, identification of molecules that regulate their function has practical significance. miRNAs and lncRNAs have been shown to influence activity and expansion of CSCs. The impact of these regulatory ranscripts on CSCs has been mostly assessed in breast cancer and HCC.

A number of studies have demonstrated the role of CSC-related miRNAs/lncRNAs such as miR-1976, miR-196a-5p, miR-221, HOTAIR, and GAS5 in EMT, emphasizing on the multifaceted functions of these transcripts in the carcinogenesis and connection between these cancer-related processes. The impact of CSC-related miRNAs/lncRNAs on radio/chemoresistance has also been assessed. miR-142-3p, miR-375, miR-494, miR-223, miR-132, miR-185, miR-181b, TP73-AS1 and GAS5 are among non-coding RNAs with appreciated roles in this aspect. Notably, miRNAs/lncRNAs that attenuate expansion of CSCs cab have synergic effects with conventional anti-cancer drugs, therefore these targeted therapies migh have promisisng effects in the clinical settings.

Among lncRNAs, MALAT1, HOTAIR, and XIST have been the mostly assessed ones in CSCs. Independent studies have verified the effects of these lncRNAs in expansion of CSCs. The effects of lncRNAs in modulation of function of CSCs are mediated through different routes, among them is their miRNA spoging function. MUF/miR-34a, MALAT1/miR-375, XIST/miR-152, XIST/miR-200c,

GAS5/miR-221, lnc-ROR/miR-145, BCAR4/miR-665, MBNL1-AS1/miR-412-3p, PVT1/miR-1207, CCAT1/miR-185-3p, DOCK9-AS2/miR-1972 and LHFPL3-AS1/miR-181a-5p are examples of lncRNAs/miRNAs axes which contribute in the regulation of CSCs.

miRNAs can simultaneously regulate CSC-related pathways such as Wnt/β-catenin signaling through targeting multiple regulators of these pathways. miR-92a (Zhang G.-J. et al., 2017), miR-106b-5p (Lu et al., 2017b) and miR-1275 (Jiang et al., 2020) are examples of miRNAs with this kind of aptitude. Identification of additional miRNAs with the ability to target CSC-related pathways at multiple levels would facilitate enhancing efficiency of therapeutic interventions.

Wnt/β-catenin pathway as one of the most important pathways in determination of stem cells fate is regulated by several miRNAs such as miR-600, miR-142-3p, miR-31, miR-217, miR-92a, miR-150-5p, miR-106b-5p, miR-1275, miR-708-5p, miR-1301-3p as well as a number of lncRNAs such as lncTCF7, MUF, HOTTIP and DOCK9-AS2. Activity of Notch pathway is modulated by miR-7-5p, miR-26a, miR-1275, and miR-181b.

The underlying cause of abnormal expression of these non-coding RNAs has not been clarified completely. However, methylation marks in the promoter region can explain down-regulation of a number of these non-coding RNAs. miR-7-5p is an exmaple of these non-coding RNAs (Xin et al., 2020). Meanwhile, some lncRNAs have pivotal roels in the modulation of methylation of target genes. Therefore, a complicated interaction network exists between epigenetic factors that regulate methylation marks, miRNAs, lncRNAs and transcription factors that modulate expression of lncRNAs/miRNAs or are regulated by these transcripts. Understanding this multifaceted network is the prerequisite of design of targeted therapies against CSCs.

Taken together, several miRNAs and lncRNAs have been identified that regulate expansion of CSCs via different mechanisms, particularly regulation of cancer-related signaling pathways. Based on the importance of CSCs in the metastatic ability of malignnat cells and their resistance to therapeutic regimens, these transcripts represent promising targets in cancer management. Most of accomplished studies have assessed the impact of lncRNA/miRNA silencing or up-regulation in cell lines. However, a number of eminnet studies in this field have validated the results of *in silico* and *in vitro* studies in animal models of cancers providing more valuable clues for implementation of these methods in clinical settings. miR-500a-3p, miR-92a and XIST are among non-coding RNAs with sufficient *in vivo* studies.

## Author Contributions

MT and SG-F wrote the draft and revised it. HS, ZB, MH, and GS collected the data and designed the tables and study. All the authors read and approved submitted version.

## Conflict of Interest

The authors declare that the research was conducted in the absence of any commercial or financial relationships that could be construed as a potential conflict of interest.
